# Microbiome-Derived Lipopolysaccharide (LPS) Selectively Inhibits Neurofilament Light Chain (NF-L) Gene Expression in Human Neuronal-Glial (HNG) Cells in Primary Culture

**DOI:** 10.3389/fnins.2018.00896

**Published:** 2018-12-05

**Authors:** Walter J. Lukiw, Lin Cong, Vivian Jaber, Yuhai Zhao

**Affiliations:** ^1^Neuroscience Center, Louisiana State University School of Medicine, Louisiana State University Health Sciences Center, New Orleans, LA, United States; ^2^Department of Neurology, Louisiana State University School of Medicine, Louisiana State University Health Sciences Center, New Orleans, LA, United States; ^3^Department of Ophthalmology, Louisiana State University School of Medicine, Louisiana State University Health Sciences Center, New Orleans, LA, United States; ^4^Department of Neurology, Shengjing Hospital, China Medical University, Shenyang, China; ^5^Department of Anatomy and Cell Biology, Louisiana State University School of Medicine, Louisiana State University Health Sciences Center, New Orleans, LA, United States

**Keywords:** Alzheimer’s disease (AD), DNA array, inflammatory degeneration, lipopolysaccharide (LPS), microbiome, neurofilament light chain (NF-L), neurofilament triplet

## Abstract

The remarkable co-localization of highly pro-inflammatory lipopolysaccharide (LPS) with sporadic Alzheimer’s disease (AD)-affected neuronal nuclei suggests that there may be some novel pathogenic contribution of this heat stable neurotoxin to neuronal activity and neuron-specific gene expression. In this communication we show for the first time: (i) the association and envelopment of sporadic AD neuronal nuclei with LPS in multiple AD neocortical tissue samples; and (ii) a selective repression in the output of neuron-specific neurofilament light (NF-L) chain messenger RNA (mRNA), perhaps as a consequence of this association. The down-regulation of NF-L mRNA and protein is a characteristic attribute of AD brain and accompanies neuronal atrophy and an associated loss of neuronal architecture with synaptic deficits. To study this phenomenon further, human neuronal-glial (HNG) cells in primary culture were incubated with LPS, and DNA arrays, Northern, Western, and ELISA analyses were used to quantify transcription patterns for the three member neuron-specific intermediate filament-gene family NF-H, NF-M, and NF-L. As in sporadic AD limbic-regions, down-regulated transcription products for the NF-L intermediate filament protein was significant. These results support our novel hypothesis: (i) that internally sourced, microbiome-derived neurotoxins such as LPS contribute to a progressive disruption in the read-out of neuron-specific genetic-information; (ii) that the presence of LPS-enveloped neuronal nuclei is associated with a down-regulation in NF-L expression, a key neuron-specific cytoskeletal component; and (iii) this may have a bearing on progressive neuronal atrophy, loss of synaptic-contact and disruption of neuronal architecture, all of which are characteristic pathological features of sporadic-AD brain. This is the first report that provides evidence for a neuron-specific effect of a human GI-tract microbiome-derived neurotoxin on decreased NF-L abundance in both sporadic AD temporal lobe neocortex *in vivo* and in LPS-stressed HNG cells *in vitro*.

## Introduction

Recently there has been a resurgence of interest in the human gastrointestinal (GI) tract microbiome and its potential contribution to human health and disease. One area receiving considerable research attention has been the possible involvement of human GI-tract microbiome-derived neurotoxins with progressive and terminal neurological diseases associated with aging and inflammatory neurodegeneration. These microbiome-derived neurotoxic exudates consist of a remarkably complex and neurobiologically potent array of pro-inflammatory endotoxins and exotoxins (such as fagilysin), lipooligosaccharides (LOS), lipopolysaccharides (LPS; including the extremely pro-inflammatory *B. fragilis* LPS, BF-LPS), microRNA-like small non-coding RNAs (sncRNA), and an extensive variety of bacterial-derived amyloids (Hofer, 2014; Foster et al., 2016; Lukiw, 2016a,b; Zhan et al., 2016, 2018; Mancuso and Santangelo, 2017; Yang and Chiu, 2017; Zhao et al., 2017a,b,c; Zhao and Lukiw, 2018). Several recent papers have addressed the emerging link between elements of the human GI-tract microbiome and Alzheimer’s disease (AD), a common, chronic, and progressive age-related neurodegenerative disease whose incidence is reaching epidemic proportions and represents a major, lethal, neuropsychiatric disorder that currently constitutes a major healthcare concern worldwide (Zhan et al., 2016; Jiang et al., 2017; Cox and Weiner, 2018; Szablewski, 2018; Zhao and Lukiw, 2018).

Both the familial and the much more common sporadic forms of AD are characterized by the appearance of extracellular deposits including dense, insoluble amyloid-beta (Aβ) peptide enriched senile plaques (SP) and tau- and neurofilament-protein enriched neurofibrillary tangles (NFT), and neuropathologically by the progressive atrophy of large neurons, ensuing loss of synaptic contacts and altered neuronal cytoarchitecture (Clark and Vissel, 2015; Zhao and Lukiw, 2015; Minter et al., 2016). We adopted the strategy that because inter-synaptic connections, the radial diameter of neurons and the overall neuronal architecture and morphology are maintained in large part by this relatively abundant three member neuron-specific neurofilament gene family – encoding the neurofilament light (NF-L; NEFL; 68 kDa), neurofilament medium (NF-M; ∼160 kDa), and neurofilament heavy (NF-H; ∼205 kDa) chain proteins – we reasoned that NF-L, NF-M, and NF-H relative abundance would be an experimentally practical and suitable choice to look for changes in expression in both sporadic AD brain and in LPS treated human neuronal-glial (HNG) cells in primary culture.

Our findings indicate for the first time, that linked to a progressive association of the amphiphilic glycolipid LPS with sporadic AD neuronal nuclei there appears to be a parallel and selective repression in the output of neuron-specific NF-L mRNA in AD brain compared to age-and gender-matched controls. This is noteworthy because down-regulation of NF-L expression is a characteristic feature of the limbic system in AD and accompanies the atrophy of neurons and progressive loss of neuronal architecture and synaptic contact in the AD brain (McLachlan et al., 1988; Lukiw et al., 1990; Julien and Mushynski, 1998; Clement et al., 2016; Khalil et al., 2018). These effects were further observed in HNG primary cultures incubated with Gram-negative bacterial-derived LPS in which was observed a significant LPS-mediated down-regulation of the NF-L intermediate filament protein. Taken together these results suggest: (i) that microbiome-derived LPS may contribute to a progressive disruption in the read-out of the brain’s neuron-specific genetic-information; (ii) that NF-L mRNA and the expression of NF-L proteins are one important neuron-specific transcript targeted by microbiome-derived LPS; and (iii) that this may have a bearing on neuronal atrophy, disruption of the neuronal architecture and loss of synaptic organization, all of which are characteristic neuropathological features of AD-affected brain.

## Materials and Methods

### Human Brain Tissues, Antibodies and Immunohistochemistry

Female control [*N* = 12; mean age ± one standard deviation of 85.8 ± 2.1 years with a post-mortem interval (PMI) of (mean ± one standard deviation) 3.6 ± 1.5 h] and age-matched AD (*N* = 12; age 87.7 ± 2.5 years and PMI 3.8 ± 1.2 h) human superior temporal lobe neocortical tissues (Brodmann A22) were obtained from the University of Maryland, from archived material at the University of Toronto and the Louisiana State University Neuroscience Center, and the University of California (UC)-Irvine Brain Bank. A total of 24 female, age-, gender-, and PMI-matched control and AD brains were examined for LPS content. For immunocytochemistry of LPS human brain tissue samples were embedded in OCT and frozen at -80°C; 10 μm brain sections were cut using a Shandon cryotome (Waltham, MA, United States). After an initial fixation with 4% paraformaldehyde for 20 min, sections were then incubated in primary antibodies (1:1000; 1 × PBS with 2% BSA, 2% goat or donkey serum and 0.1% TX-100) overnight at 4°C, washed with PBS, and then incubated with Alexa Fluor-conjugated species-specific secondary antibodies (ThermoFisher Scientific, Waltham, MA, United States) for 3 h at RT (see further details below). Sections were counter-stained with DAPI for nuclei, followed by quenching with Autofluorescence Eliminator Reagent (Millipore Cat No. 2160; Zhan et al., 2016; Zhao et al., 2017a,b,c), mounted on glass slides, cover-slipped with Fluoromount-G (ThermoFisher Scientific) and imaged using a Zeiss LSM 700 Confocal Laser Scanning microscope system (Carl Zeiss Microscopy, Thornwood, NY, United States; Bagyinszky et al., 2017; Zhao et al., 2017a,b,c^1^).

#### Human Neuronal-Glial (HNG) Cells in Primary Co-culture

Human neuronal-glial primary cells, cryopreserved at first passage one, were obtained from commercial sources and cultured according to the manufacturer’s instructions (Lonza PT-2599, Lonza Cell Systems, Allendale, NJ, United States or Cell Systems, ACBRI 376, Kirkland, WA, United States). HNG cells tested negative for HIV-1, HBV, HCV, mycoplasma, bacteria, yeast, and fungi at source, and have been extensively used for studies on brain gene induction and gene expression, neuronal development, neurotoxicology, neuropharmacology, and in *in vitro* models of AD and other age-related neurological disorders that exhibit a progressive age-related inflammatory neurodegeneration (Li et al., 2011; Zhao et al., 2017a,b,c; Zhao and Lukiw, 2018). HNG cells demonstrate particular neuronal and astroglial cell markers including neuron-specific β-tubulin III (βtubIII; red staining; λ_max_ = 690 nm) and glial fibrillary acidic protein (GFAP; glial-specific green stain; λ_max_ = 520 nm). Briefly, HNG cells were maintained as free-floating aggregates (neurospheres) in 75 cm^2^ uncoated plastic flask in neural progenitor maintenance media (NPMM; Lonza CC-3209) supplemented with recombinant human fibroblast growth factor (rhFGF) and epidermal growth factor [rhEGF]) and neural survival factor-1 [NSF-1] (Lonza CC-4242) and gentamicin/amphotericin-B (Lonza GA-1000). Differentiation was induced by plating neurospheres onto eight-well glass chamber-slides pre-coated with poly-L-ornithine (an amino acid polymer used as substratum to improve neuronal adhesion); cells were kept at 37°C in a humidified 5% CO_2_ atmosphere incubator at all times. The differentiation media (Lonza CC-4242) was free of growth factors but contained NSF and gentamicin/amphotericin-B, 25 ng/ml of brain-derived neurotrophic factor (BDNF), and 1% of fetal bovine serum (FBS). Upon deprivation of growth factors neurospheres began to attach to the well bottoms and next migrated out to form a co-culture of human neurons and glial cells (HNG). HNG cells were used 2 weeks after induction of differentiation; HNG cells initially contained about 5 × 10^5^ cells/ml volume and were cultured to ∼70% confluency in HNG cell medium as described in detail (Cui et al., 2010; Bhattacharjee and Lukiw, 2013; Zhao et al., 2014, 2017a,b,c; Lukiw, 2016a,b). HNG cells were subsequently incubated with LPS; the concentration of LPS (Sigma L2630^2^) provided to 2-week-old HNG cells cultured in HNG cell medium was 50 nM for 48 h (see Figure 4; for further specific details see also Zhao et al., 2017a,b,c). Higher doses of LPS (up to 5 μM) in HNG cell medium for shorter periods gave comparable results (data not shown).

### Immunofluorescence Protocol

**FIGURE 1:**
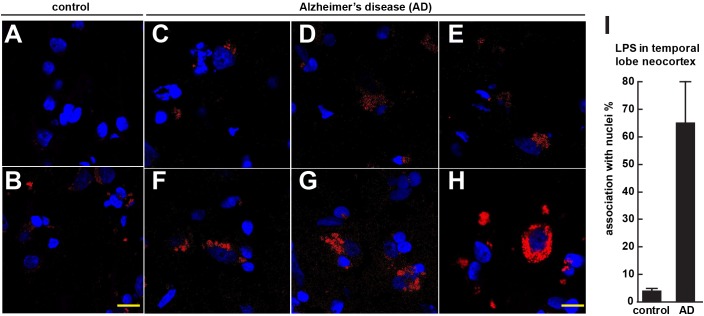
Controls **(A,B)** shows staining of human control superior temporal lobe neocortex (CDR = 0) with LPS (red stain; λ_max_ = 690 nm) and DAPI (blue stain; λ_max_ = 470 nm); Alzheimer’s disease **(C–E)** CDR = 2.0 and **(F–H)** CDR = 3.0 illustrates increasing presence of LPS and association of superior temporal lobe AD neocortical nuclei with LPS; LPS staining was observed in controls but the staining was relatively rare and punctate compared to LPS in AD **(C–H)** with the observation of the eventual encapsulation of nuclei by LPS **(F)**; (see text); the bar graph in **(I)** quantifies LPS association with nuclei in the temporal lobe neocortex (see text and Zhao et al., 2017a,b); to ascertain whether LPS-associated and LPS-encapsulated AD nuclei were of neuronal or astroglial origin we next counterstained with the neuron-specific green stain NeuN (λ_max_ = 520 nm; see Figure 2); magnification 63 × ; scale bar = 20 μm.

**FIGURE 2:**
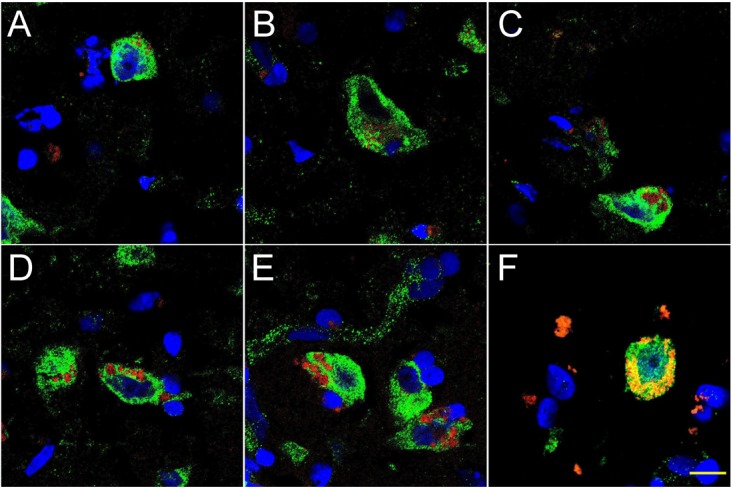
Progressive association and envelopment of AD-affected neocortical neuronal nuclei by LPS (red stain; λ_max_ = 690 nm), DAPI (blue nuclear stain; λ_max_ = 470 nm) and NeuN (neuron-specific green stain; λ_max_ = 520 nm); human superior temporal lobe AD neocortex (Brodmann A22) from CDR (clinical dementia rating) 1.0, 2.0 and 3.0 AD brains; **A,B** = CDR 1.0; **C,D** = CDR 2.0; **E,F** = CDR 3.0 (see also https://knightadrc.wustl.edu/cdr/cdr.htm); LPS staining (red) was subjected to co-localization analysis with the neuronal marker NeuN (green) and/or nuclear marker (blue); magnification 63×; scale bar = 20 μm.

**FIGURE 3:**
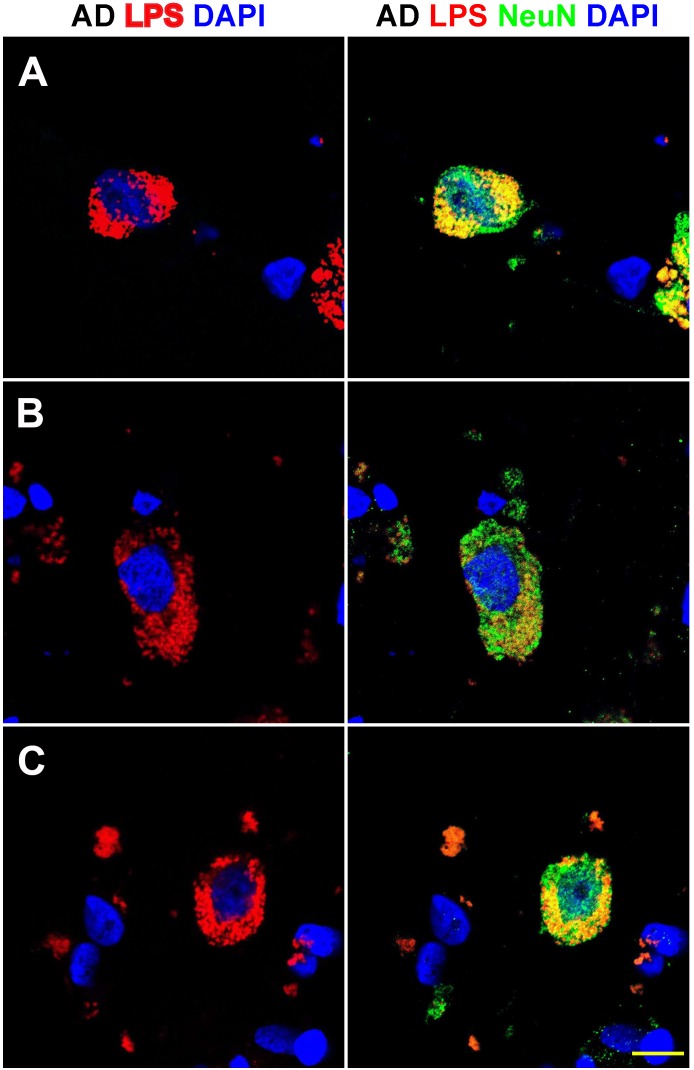
Details of the perinuclear association and envelopment of multiple neuronal nuclei by LPS in sporadic Alzheimer’s disease (AD) brain (Brodmann Area 22; superior temporal lobe neocortex); **(A–C)** details of LPS association with neuronal nuclei in three AD independent brain samples; all three AD brain samples were from moderate-to-advanced-AD (CDR 2.0-3.0); no such extensive association between LPS and control brain nuclei was observed (Figure 1); lipopolysaccharide (LPS; red stain; λmax = 690 nm); DAPI (nuclear-specific blue stain; λmax = 470 nm); NeuN (neuron-specific green stain; λmax = 520 nm); brain sections are 10 μm thick (see text); a large number of neuronal nuclei in sporadic AD were found to be completely enveloped by LPS especially in the later stages of sporadic AD (CDR 3.0); scale bar for all photos here (lower right) = 20 μm.

Two week old cultures of HNG cells in eight-well chamber slides (BD Biosciences, San Jose, CA, United States) were fixed with 4% paraformaldehyde, then permeabilized and blocked with 0.125% Triton X-100 and 2% normal goat serum in PBS at RT for 1 h. Cells were incubated overnight at 4°C with antibodies for β-tubulin III (for neurons; Sigma T8578, Sigma-Aldrich St. Louis, MO, United States) and GFAP (for astrocytes; Sigma G9629). Cells were subsequently washed for three times with PBS and then incubated for 3 h at room temperature with secondary antibodies conjugated with cy3 or FITC fluorescein (Thermofisher A21422 and A11008; ThermoFisher Scientific, Waltham, MA, United States). After washing and drying, slides were applied with mounting medium containing DAPI (1:10,000; Vector Laboratories, Burlingame, CA, United States) and observed under Zeiss Axioplan Inverted Deconvolution Fluorescent Microscope (63× oil immersion lens; Carl Zeiss, Oberkochen, Germany). Positively stained cells were quantified manually using the manual counter function of ImageJ software (NIH). Negative control with quenching was performed as previously reported in detail (Zhao et al., 2017a,b,c); quantification of LPS was analyzed (i) as a percentage of neuronal area (see below); and/or (ii) by counting multiple microscope fields for the quantity of LPS signals (red stain; λ_max_ = 690 nm) associated with DAPI (blue nuclear stain; λ_max_ = 470 nm) (see Figures 1–3).

### Antibodies – Specificity and Validation

We used mouse anti-*E. coli* LPS (Abcam Cat No. ab35654; Cambridge, MA, United States); rabbit anti-NeuN (Cell Signaling, Cat No. 24307), rabbit anti-GFAP (Sigma-Aldrich, Cat No. G4564) (Cui et al., 2010; Zhao et al., 2014; Lukiw, 2016a, 2017; Zhan et al., 2016). LPS antibody specificity and validation was confirmed (i) using Western immunoblot analysis (see Figure 1 in Zhao et al., 2017a) which corresponded to the manufacturer’s published specifications^3^; and (ii) an antibody neutralization/LPS quenching control assay (Zhao et al., 2017a,b,c). We used sandwich ELISA and Western analysis for NF-L protein determination in LPS-treated HNG cells using Abbexa (abx250460; Cambridge, United Kingdom) and/or LifeSpan BioSciences (LSBio; LS-F6701; Seattle, WA, United States) ELISA systems and NF-L (NEFL) monoclonal antibody (DA2; ThermoFisher Scientific, Cat No. MA1-2010) and a beta actin (β-actin) loading control monoclonal antibody (BA3R; ThermoFisher Scientific, Cat No. MA5-15739) and standard Western analysis as has been previously described by our laboratory (Zhao et al., 2017a,c). To ascertain the association of LPS with neuronal cells confocal images of LPS and NeuN staining were imported into ImageJ^4^; RGB images were first converted into images of separate channels (red for LPS; green for NeuN; and blue for DAPI-stained nuclei). A co-localization finder plugin was run to generate images of co-localization of both channels; each co-localization image was converted into an 8-bit image and inverted. Global thresholding was utilized and the cutoff value was adjusted to the point that only highlighted co-localized particles are black on the image against a white background. Particle analysis was next performed to calculate the area size of the co-localization; this value was then divided by the area size of the NeuN or DAPI staining as the percentage of cell area (Zhao et al., 2017a,b).

### RNA Isolation and Purification, DNA Array, Northern, ELISA, and Western Analysis

**FIGURE 4:**
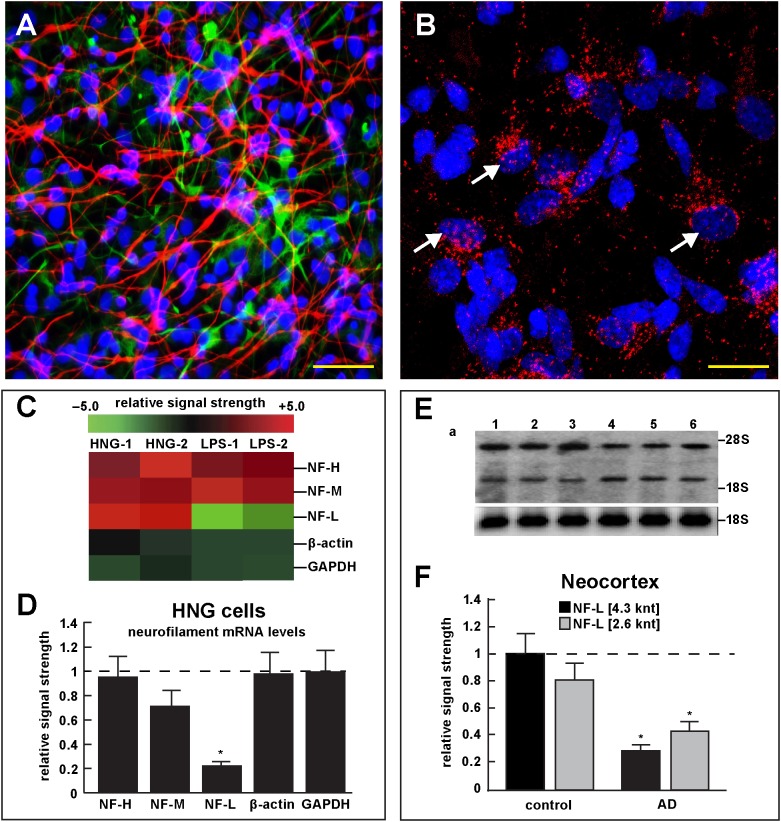
Studies of LPS-neuronal nuclear binding in HNG cells in primary culture. **(A)** human neuronal-glial (HNG) cells in primary co-culture at 2 weeks; neurons (red stain; λ_max_ = 690 nm), DAPI (blue nuclear stain; λ_max_ = 470 nm) and GFAP (glial-specific green stain; λ_max_ = 520 nm); human neurons do not culture well in the absence of glia; neurons also show both extensive arborization and display electrical activity (unpublished; Lonza); scale bar = 20 μm; **(B)** details of association of LPS (red stain; λ_max_ = 690 nm) and nuclear DAPI (blue stain; λ_max_ = 470 nm); note affinity of red-stained LPS with blue-stained nuclei after only 48 h of co-incubation (arrows); see also Supplementary File 1 (Details of accumulation of LPS in HNG cells in primary culture); scale bar for all photos (lower right) = 10 μm; **(C)** Neurofilament heavy, medium and light (NF-H, NF-M, and NF-L) chain abundance in control and LPS-treated HNG cells – cluster analysis of gene expression (mRNA levels); in two controls (HNG-1 and HNG-2) and in two LPS-treated samples (LPS-1, LPS-2), LPS-treated HNG cells exhibit a marked reduction in NF-L expression, a reduction that is not as apparent in NF-H or NF-M expression; NF-H, NF-M, and NF-L expression was quantified against the levels of β-actin and GAPDH in the same sample; **(D)** samples are quantified in bar graph format showing the mean and one standard deviation of all three neurofilament protein levels; there was no statistically significant change in NF-H, NF-M, β-actin, or GAPDH between control and LPS-treated HNG cells, however NF-L levels were reduced to about 0.22-fold of controls in LPS-treated HNG cells; interestingly the NF-H, NF-M, and NF-L mRNAs encode intermediate filaments of ∼60, ∼100, and ∼110 kDa, respectively, but due to extensive post-translational modifications such as phosphorylation and glycosylation, NF-H, NF-M, and NF-L exhibit higher molecular weights after SDS-PAGE (Western) analysis of ∼68, ∼160, and ∼205 kDa, respectively; a dashed horizontal line at 1.0 is included for ease of comparison; *N* = 3 to 5 experiments for each treatment; ^∗^*p* < 0.01 (ANOVA); **(E)** Northern blot analysis – decreased NF-L in AD – Northern analysis of total NF-L mRNA in control (lanes 1–3) and AD (lanes 4–6) temporal lobe neocortex (Brodmann A22); the position of the migration of 28S and 18S RNA (4.7 and 1.9 knt, respectively) are marked on the right of the gel (upper panel); the size of the two prominent NF-L mRNA bands detected are respectively about 4.3 and 2.6 knt in length; an 18S RNA was used as an internal control marker (lower panel); **(F)** Northern blots were quantified in bar graph format showing the mean and one standard deviation of decreased NF-L mRNA signals in AD neocortex versus age-matched controls; in AD the 2 NF-L bands [between the 28S and 18S RNA markers of part **(E)**] together are about 0.3- to 0.4-fold AD over control; ^∗^*p* < 0.01 (ANOVA).

Ultrapure chemicals and reagents of the highest grades commercially available were used throughout these experiments. Typically, 10 mM phenylmethylsulfonyl fluoride (PMSF; Sigma) and 1 U human placenta ribonuclease inhibitor (RNasin; Promega Corporation, Madison WI, United States) were employed in the extraction medium to inhibit protease and specific ribonuclease activities in homogenized human brain tissues or HNG cells. Total cellular RNA was isolated using TRIzol Reagent (Invitrogen-ThermoFisher Scientific; Cat No. 15596026) and quality controlled using analysis using and Agilent 2100 bioanalyzer (Agilent Technologies, Santa Clara CA, United States). For Northern blots ∼15 μg of total RNA was separated at 4°C on 1.5% agarose/2.2 M formaldehyde gels at 60 V for 15 h with recirculating 20 mM sodium phosphate buffer, pH 7.0. Gels were stained for 10 min with acridine orange, visualized at 340 nm on a UV trans-illuminator and total RNA was blotted onto Biotrans 0.2 μm Biotrans nylon membrane (Cat No. 01811300, MP Biomedicals). NF-H, NF-M, and NF-L probes were prepared to specific activities of 10^8^ dpm ^32^p-labeled dCTP per/μg of DNA as previously described in detail (Clement et al., 2016). Membranes were pre-hybridized for 12 h at 42°C in 50% formamide, 5× Denhardt’s solution (containing 0.1% each of Ficoll 400, polyvinylpyrrolidone, and bovine serum albumin), 5× SSC (standard saline citrate, containing 150 mM sodium chloride, 15 mM sodium citrate, pH 7.0), 50 mM sodium phosphate buffer, pH 6.5, 0.1% sodium dodecyl sulfate (SDS), and 350 μg/ml sonicated herring sperm. This was replaced with fresh hybridization solution containing approximately 5 × 10^7^ cpm of heat-denatured cDNA probes and hybridization occurred at 42°C for 30 h. Nylon membranes were washed under conditions of high stringency (two 30-min washes at 2 × SSC/0.1% SDS at room temperature; two 60-min washes at 0.1× SSC/0.5% SDS at 60–65°C and finally two 15-min washes at 1 × SSC/0.1% SDS at room temperature). When used Fuji RX film was exposed at -70°C for 18–72 h using standard autoradiographic imaging techniques; alternately hybridization signals were quantified using a Typhoon FLA 9500 Biomolecular Imager (GE Healthcare). DNA array analysis was performed as extensively described by our group (Colangelo et al., 2002; Clement et al., 2016; Jaber et al., 2017). Sandwich ELISA and/or Western analysis was used for NF-L protein abundance analysis according to the manufacturers’ instructions to quantify both NF-L and β-actin control abundance levels (Figure 4).

### Statistical Analysis, Integrated Bioinformatics Analysis, and Data Interpretation

For NF-H, NF-M, NF-L, β-actin, and GAPDH mRNA abundance analysis all statistical procedures were analyzed using *p*, analysis of variance (ANOVA) a two-way factorial analysis of variance using algorithms, and/or procedures in the SAS language (Statistical Analysis Institute, Cary, NC, United States) and as previously described (Cui et al., 2010; Zhao et al., 2011; Clement et al., 2016; Dendooven and Luisi, 2017; Zhao and Lukiw, 2018). In the results *p*-values of less than 0.05 (ANOVA) were considered to be statistically significant. All NF-H, NF-M, NF-L, β-actin, and GAPDH mRNA abundance data were collected and analyzed using Excel 2016 (Office 365) algorithms (Microsoft Corporation, Redmond WA, United States); all figures were generated using Adobe Illustrator CC 2015 and Photoshop CC version14.0 (Adobe Corporation, San Jose, CA, United States).

## Results

Figure 1 (control A,B) shows staining of 10 μm brain sections of human control superior temporal lobe neocortex (Brodmann A22; CDR = 0) of Figure 1A an 86 year-old female, 3.6 h PMI and Figure 1B an 85-year-old female, 4.3 h PMI) stained with LPS (red stain; λ_max_ = 690 nm) and DAPI (blue stain; λ_max_ = 470 nm); control samples (A) and (B) were from patients with no history of neurodegenerative disease or cognitive impairment; LPS staining was infrequently observed in controls and the staining was sparse; Alzheimer’s disease (Figures 1C–H); (Figures 1C–E) CDR = 2.0; and (Figures 1F–H) CDR = 3.0; all female; age-range 79–89; PMI range 3.3–4.1 h; illustrates progressive association and envelopment of AD neocortical nuclei with LPS–LPS (red stain; λ_max_ = 690 nm) and DAPI (blue stain; λ_max_ = 470 nm) staining of human superior temporal lobe AD neocortex (Brodmann A22); note minor ‘punctate’ LPS signals in control (Figures 1A,B) not associated with nuclei, compared to LPS in AD (Figures 1C–F) and encapsulation of nuclei by LPS. In a previous study LPS was reported to range from a ∼7- to ∼21-fold increase in abundance in AD brain over age-matched controls from the same anatomical region (Zhao et al., 2017a). The bar graph in Figure 1I quantifies LPS association with nuclei in the temporal lobe neocortex (see text); in AD over control; this ratio is about 16.3 for the samples examined; to investigate whether LPS-encapsulated AD nuclei were of neuronal or astroglial origin we next counterstained with the neuron-specific green stain NeuN (Figure 2).

Figure 2 shows 10 μM sections of AD neocortical brain tissue stained additionally with NeuN, a neuron-specific green stain (λ_max_ = 520 nm). Interestingly, (i) all LPS staining appears to be confined to one specific nuclear region of AD neuronal nuclei; and (ii) the entire neuronal perinuclear region was occupied by LPS stain in about 5–15% of all neuronal nuclei associated with LPS especially in the later stages of sporadic AD (CDR = 3.0).

Figure 3 shows detail of this LPS-AD neuronal nuclear interaction; approximately 60–70% of all moderate-to-late-stage (CDR 2.0 to 3.0) AD neocortical neuronal nuclei exhibited an association with LPS and about 5–15% of all AD neuronal nuclei in the temporal lobe neocortex showed a complete envelopment by LPS in the 12 sporadic AD cases investigate in this study. Immunocytochemistry further indicated that the “thickness” of “perinuclear LPS envelopes” ranged between 2 and 20 μm (Figures 2, 3).

Figure 4 describes experiments in human neuronal-glial (HNG) cells in primary culture exposed to LPS; HNG cells (Figure 4A) cultured for 2 weeks, 60% confluent and containing about 70% neurons and 30% astroglia, were exposed to 50 nM LPS for 48 h, total RNA was isolated and analyzed on DNA arrays as extensively described by our laboratories (Colangelo et al., 2002; Clement et al., 2016; Jaber et al., 2017). Even at brief periods of exposure to LPS (48 h), LPS appeared to have a strong affinity for DAPI-stained neocortical nuclei (Figure 4B). DNA array analysis (Figure 4C) indicated that in comparison to the DNA array control transcripts β-actin and glyceraldehyde phosphate dehydrogenase (GAPDH), NF-H mRNA, NF-M mRNA, and NF-L mRNA were found to be reduced to 0.95- 0.72-, and 0.25-fold of controls; in these experiments the NF-L mRNA reduced to 0.25-fold of controls achieved the highest significance of down-regulation (*p* < 0.01, ANOVA); Figure 4D represents the quantification of these signals in bar-graph format. Figure 4E shows the results of a Northern blot indicating decreased abundance of NF-L mRNA in AD versus age- and gender-matched controls and Figure 4F shows the quantified results in bar graph format comparing both the 4.3 and 2.6 knt NF-L mRNA abundance in control and AD. For additional details, please refer to the legend to Figure 4.

**FIGURE 5:**
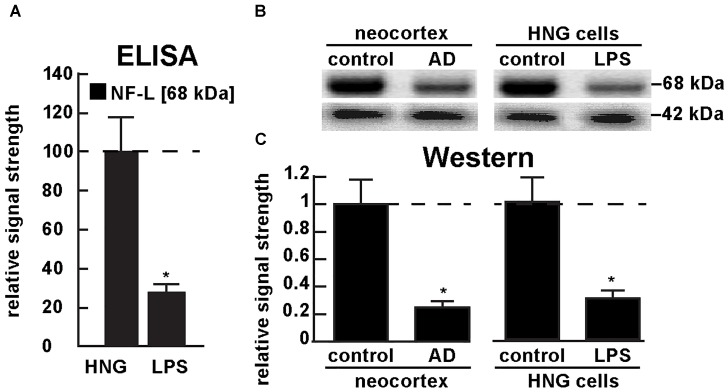
Decreased NF-L protein in LPS-treated HNG cells and in AD: ELISA and Western analysis. **(A)** results of sandwich ELISA analysis for NF-L protein in LPS-treated HNG cells using Abbexa (abx250460; Cambridge, United Kingdom) and/or LifeSpan BioSciences (LSBio; LS-F6701; Seattle WA, United States); the 68 kDa NF-L species is a particularly abundant intermediate filament protein, however in the presence of LPS the abundance of NF-L protein was found to be reduced to about 0.3-fold of control; a dashed horizontal line at 100 is included for ease of comparison; *N* = 3 to 5 experiments per determination; ^∗^*p* < 0.01 (ANOVA); **(B)** Western analysis of total NF-L protein (MW ∼68 kDa) in control (pool of five controls and five AD temporal lobe neocortex Brodman A22) and total NF-L protein in control and LPS-treated HNG cells (at 2 weeks of culture; see Figure 4A); β-actin protein (MW ∼42 kDa) was used as an internal control marker in the same sample for each determination; **(C)** Western blots were quantified in bar graph format of decreased NF-L protein abundance in AD neocortex versus age-matched controls and in LPS-treated HNG cells versus age-matched controls; a dashed horizontal line at 1.0 is included for ease of comparison; the results of decreased NF-L expression for AD over control or LPS-treated HNG cells over control are highly significant; *N* = 3 to 5 experiments; ^∗^*p* < 0.01 (ANOVA).

Figure 5A shows the results of a sandwich ELISA analysis confirming decreased abundance of NF-L protein in AD versus age- and gender-matched controls to about 0.3-fold of control. Figure 5B shows the results of a Western analysis of total NF-L protein (MW ∼68 kDa) in control and AD neocortex (left panel) and control and LPS-treated HNG cells (right panel) using β-actin (MW ∼42 kDa) as an internal control and gel-loading marker. Figure 5C shows the quantified results in bar graph format comparing NF-L protein abundance in AD and in age- and gender-matched control and in control and LPS-treated HNG cells. Therefore, the independent techniques of ELISA and Western analysis corroborated the observation that the normally highly abundant NF-L protein is reduced in both LPS-enriched AD-affected brain *in vivo* and in LPS-treated HNG cells *in vitro*. For additional experimental details please refer to the legend of Figure 5.

## Discussion

### Gastrointestinal (GI) Tract Microbiome-Derived Neurotoxins in Neurological Disease

The human GI tract, containing about 95% of the entire human microbiome, is the largest reservoir of microorganisms in the human body (Bhattacharjee and Lukiw, 2013; Zhao and Lukiw, 2015; Zhan et al., 2016; Vogt et al., 2017; Zhao et al., 2017a; Zhao and Lukiw, 2018). Comprised of a genetically diverse and densely packed repository of about 100 trillion microorganisms, the GI-tract microbiome is made up of mostly of anaerobic bacterial species with archaebacteria, fungi, protozoa, viruses, and other microbes making up the remainder (Bhattacharjee and Lukiw, 2013; Köhler et al., 2016; Vogt et al., 2017; Zhao et al., 2017a,b,c; Zhao and Lukiw, 2018). Microbial abundance, complexity and speciation, their biophysics, microbiology and neurobiology, molecular genetics, epigenetics, the signaling mechanisms, and pathways involved in microbiome-host communications and interactions are becoming increasingly understood in context of their dynamic contribution to human neurobiology in health, aging, and disease (Bhattacharjee and Lukiw, 2013; Foster et al., 2016; Köhler et al., 2016; Jiang et al., 2017; Cox and Weiner, 2018).

GI-tract derived neurotoxins include an extraordinarily complex mixture of potentially pathogenic amyloid, exotoxins and endotoxins, lipooligosaccahrides (LOS), LPS, and small miRNA-like non-coding RNA (sncRNA) exudates. Normally confined within the healthy human GI-tract, accompanying aging, and disease these secreted neurotoxins can transverse normally protective biophysical and physiological barriers resulting in a persistent systemic inflammatory condition (Jiang et al., 2017; Magalhães et al., 2017). Leakage of GI-tract neurotoxins into the systemic circulation may be a biomarker and an early indicator of progressive, age-related neuroinflammatory disorders that include AD (Lukiw, 2017; Magalhães et al., 2017; Montagne et al., 2017; Cox and Weiner, 2018; Ho et al., 2018; Sweeney et al., 2018). Indeed progressive “leakage” of LPS across the GI-tract and blood brain barrier (BBB) may be a feature accompanying normal aging (Montagne et al., 2017; Sweeney et al., 2018). Certain GI-tract microbiota may actively assist the host in moderating inflammatory neurodegeneration by supporting the generation of short chain fatty acids (SCFAs) that can pass these barriers and subsequently interfere with the generation and aggregation of neurotoxic amyloid beta (Aβ) peptides (Cox and Weiner, 2018; Ho et al., 2018). The interactions between LPS and amyloid peptides in the brain parenchyma remain incompletely understood, especially their dynamics and potential association as they accumulate in parallel with aging both within the confines of the CNS and throughout the systemic circulation (Zhao et al., 2017a,b,c).

### LPS Transit Across Biophysical and Physiological Barriers Into the CNS

Recent data regarding the contribution of neurotoxic exudates of the human GI tract microbiome to the potential initiation, development, and/or progression of AD appears to be age-related, complicated, and significant. Major bacterial species of the human GI-tract microbiome such as the Gram-negative bacillus *Bacteroides fragilis* (*B. fragilis*) secrete a unusually complex array of highly pathogenic pro-inflammatory neurotoxins which, when released from the confines of a healthy GI tract, are highly toxic to neurons of the CNS and PNS. While an environmental cause for sporadic AD has often been suggested, a strong source of powerful neurotoxins already reside within our GI tract microbiome. LPS for example represents an internally generated GI tract microbiome-derived neurotoxin capable of driving AD-type change and has enormous potential to initiate and/or propagate inflammatory neurodegeneration along the gut-brain axis. Some incompletely understood aspects of the bioavailability to the CNS of GI-tract generated neurotoxins are (i) their translocation through the GI tract and BBB that involves dynamic structures which are known to become more “leaky” with aging and disease; (ii) the direct influence of endotoxins, such as fragilysin, which targets zonula adherens protein E-cadherin and cell–cell adhesion; and (iii) the molecular exchanges between the GI tract, the systemic circulation and the BBB to access the brain parenchyma (Seong et al., 2015; Zhan et al., 2016; Montagne et al., 2017; Tsou et al., 2017; Sweeney et al., 2018). To cite recent examples from the literature: (i) BF-LPS represents an internally generated GI tract microbiome-derived neurotoxin capable of driving and emulating AD-type change *in vitro* (Zhao and Lukiw, 2018); (ii) BF-LPS has enormous potential to initiate and/or propagate inflammatory neurodegeneration along the GI tract-CNS axis (Zhao et al., 2017a); and (iii) LPS has an unusually high and remarkable affinity for the periphery of neuronal nuclei of the human neocortex (Figures 1–4) (Zhao et al., 2017b).

### LPS and Perinuclear Association in Sporadic AD Brain

In middle-to-late-stage AD brain the perinuclear association of LPS in AD appears to be configured into “net-like” or “clathrin-like” lattice within the neuronal cell cytoplasm (Figures 1–4). The pyramidal neuronal nuclei of the human neocortex are among the largest known nuclei in the human CNS, often achieving diameters of 10–20 μm and occupy a large proportion of the neuronal soma, conducive to their “euchromatic” nature and extremely high rates of transcription (Colangelo et al., 2002; Clement et al., 2016). The current experimental evidence further suggests that neuronal nuclear “encasement” by LPS appears to have a deleterious effect on the free exit and/or expression of mRNAs through the nuclear pores into the cytoplasm – previous work has shown a decreased rate of abundance of neuronal-specific DNA transcripts in sporadic AD brain (Zhao et al., 2017b). Why the NF-L mRNA exhibits the greatest down-regulated of the neurofilament triad is not known; the NF-L light chain polypeptide is the most abundant member of the neuron-specific intermediate filament family triplet, is the major component of the highly dynamic and plastic neurites, synaptic structures, and constitutes the core of the neuronal cytoskeleton. NF-L expression is also the major regulator of the caliber of the neuronal axoskeleton and essential in neuronal development, regeneration, the plasticity of the neuronal cytoskeleton, and in the creation and the maintenance of neuronal cytoarchitecture (Julien and Mushynski, 1998; Braissant, 2007; Lam et al., 2017; Goldmann, 2018; Abu-Rumeileh et al., 2018). NF-L also serves in a critical “organizer” role in axons and dendrites and contains multiple phosphorylation sites for a surprisingly large number of neuronal-enriched protein kinases, including protein kinase A, protein kinase C, cyclin-dependent kinase 5, extracellular signal regulated kinase, glycogen synthase kinase-3, and stress-activated protein kinase gamma (de Leeuw et al., 2018; Goldmann, 2018). Perturbations in NF-L phosphorylation, structure and/or function are often observed in age-related human neurodegenerative diseases including amyotrophic lateral sclerosis, Parkinson’s disease and AD, and a down-regulation of NF-L mRNA and the presence of atypical insoluble twisted neurofilament deposits have long been known to be a common feature of an abnormal neurofilament network as seen in diseased brain tissues undergoing pro-inflammatory neurodegeneration (McLachlan et al., 1988; Lukiw et al., 1990; Julien and Mushynski, 1998; Abu-Rumeileh et al., 2018; Goldmann, 2018; Zhao and Lukiw, 2018). Importantly, neuron loss in AD appears not to be the reason for the observed loss in NF-L; a classic study of 22 control, non-AD dementia and AD brains indicated that the significant decrease of NF-L mRNA in AD neocortex could not be adequately accounted for by a non-specific effect of brain damage, by neuron cell loss or by neurons with neurofibrillary degeneration (McLachlan et al., 1988; Julien and Mushynski, 1998; Ginsberg et al., 2000; Zhao et al., 2017a,b).

Interestingly, there are reports that plasma levels of NF-L protein appear to be increased in AD and may be used as a reliable diagnostic marker for AD incidence and severity (Lista et al., 2017; Abu-Rumeileh et al., 2018; Hampel et al., 2018). However, the universality of this elevation and usefulness of plasma NF-L as a biomarker for AD has been recently brought into question (Lam et al., 2017; Zhou et al., 2017; Abu-Rumeileh et al., 2018). Very recently NF-L has been found to be significantly increased in the CSF of early-onset AD patients compared to younger controls; however, this change was not found in older AD groups (Lauridsen et al., 2017). These discrepancies and differential localization of neuronal- and AD-relevant molecules are reminiscent of the variation in abundance of Aβ42 peptides within AD tissues and the extracellular fluids such as the CSF that surrounds them; for example Aβ42 peptides show an inverse abundance between brain tissues and the CSF (Fagan et al., 2006; Grimmer et al., 2009). Another example is potentially pathogenic microRNAs including a pro-inflammatory miRNA-146a and several let-7 species which are differentially abundant in AD tissues versus the AD CSF (Alexandrov et al., 2012; Derkow et al., 2018). It is tempting to speculate that a differential “compartmentalization” of neuronal-associated AD-relevant molecular species may be one significant consequence of the neuropathology of the AD process.

### Important Unanswered Questions

Several fundamental questions remain concerning GI-tract microbiome exudates, their compartmentalization in the GI-tract and their potential effects on the neurobiology, neuropathology, and pathogenetics of neurodegenerative and neuropsychiatric disease. For example: does the presence of LPS and envelopment of neuronal nuclei in AD significantly affect the transcription of any other brain genes or neuron-enriched transcripts besides NF-L? Why are NF-L mRNA abundances selectively affected? Perhaps because NF-L expression is from an extremely high output gene? Are the translocation of transcripts exiting the neuronal nuclei impaired by the envelopment of nuclei by LPS as they appear to be? Does a life-long exposure to certain infectious agents and their secreted neurotoxins predispose an individual to develop AD at a later age? How do the secreted neurotoxins from the human GI-tract microbiome progressively leak across biophysical and physiological barriers to access the systemic circulation and CNS compartments? Do these secreted neurotoxins interact with amyloid-beta (Aβ) peptides that increase in parallel in the aging brain? What GI-tract bacterial-derived mixtures of neurotoxins are the most pathogenic in promoting inflammatory neuro-degeneration? Can the incidence of systemic inflammation be used as a biomarker or be of prognostic value to AD and other progressive neurodegenerative diseases? Does anaerobic Gram-negative bacilli-derived LPS interact pathologically with other toxins originating from archaebacteria, fungi, protozoa, viruses, and other GI-tract resident microbes? Is there a potential synergism in their combined neurotoxic actions toward neuronal nuclei of the human CNS? Perhaps most importantly, is it possible to devise a dietary strategy that promotes the lowering of LPS secretion and optimize life-long GI-tract microbiome and CNS health to minimize the risk of developing AD as we age? Furthering our molecular and mechanistic understanding of how individual secreted components of the GI tract microbiome – affect the PNS and CNS may uncover potential and novel strategies for GI tract-based modulation of neural function and the more efficacious clinical management of terminal, age-related neurological disease.

## Conclusion

**FIGURE 6:**
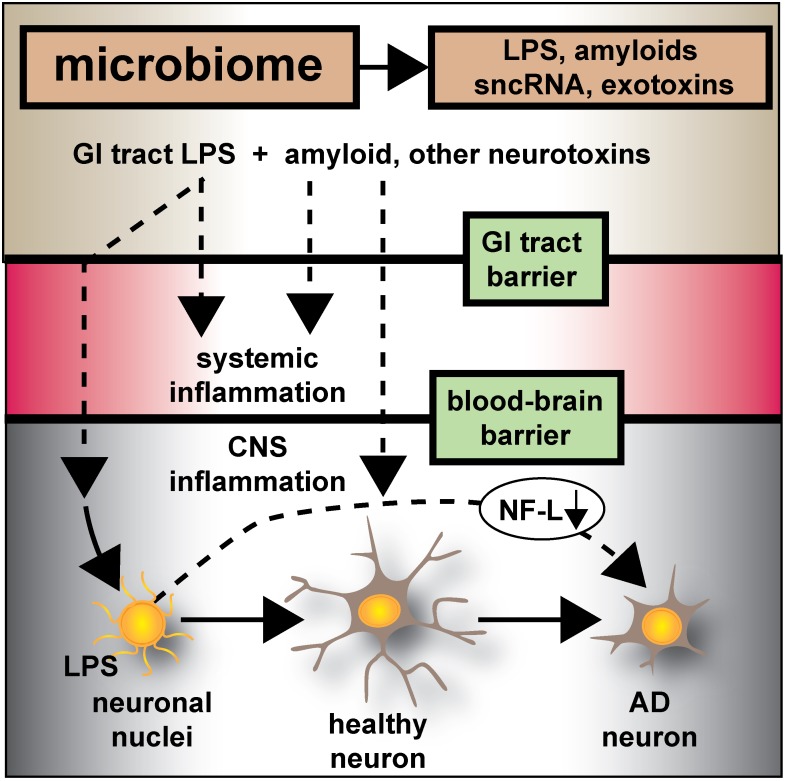
Microbiome-derived LPS-mediated impairment of NF-L expression may contribute to atrophy of neurons and cytoskeletal disorganization that is characteristic of sporadic AD – the human GI-tract microbiome secretes a remarkably heterogeneous and complex mixture of neurotoxins including different varieties of lipooligosaccahrides (LOS), lipopolysaccharide (LPS), amyloids, small non-coding RNAs (sncRNAs) and exotoxins; recently several laboratories have provided evidence that these neurotoxins may transit GI-tract and blood-brain barriers and are present in the CNS and within aged or AD brain tissues; whether these microbiome-derived neurotoxins originate from the gastrointestinal (GI) tract microbiome, a possible brain microbiome or some dormant pathological microbiome is currently not well understood. Recent studies further suggest that the co-localization of pro-inflammatory LPS with AD-affected neuronal nuclei provides evidence that there may be a contribution of LPS to genotoxic events that support deficits in homeostatic gene expression that drive progressive AD-type change and provide support for pro-inflammatory neurodegeneration. This communication provides evidence that in both LPS-enveloped neuronal nuclei in AD neocortex and LPS-treated HNG cells in primary co-culture that there is a significant deficit in the expression of the neurofilament light-chain (NF-L), a neuron-specific cytoskeletal element known to be important in maintaining the shape and synaptic integrity of the neuron; see text and Figures 1–5 for additional details.

Microbiologists, neurologists and bioinformatics researchers are still in a relatively early stage of understanding the molecular-genetic pathological signaling mechanisms that operate between the human GI-tract microbiome and the CNS of the host. Emerging evidence suggests: (i) that non-homeostatic communication along the gut-brain axis may contribute to progressive inflammatory neurodegeneration and AD-type change in the CNS; and (ii) that the GI-tract microbiome appears to have potential to contribute to neurodegenerative disease through the release, and export into the systemic circulation, of multiple, highly pro-inflammatory neurotoxic exudates predominantly from abundant species of Gram-negative anaerobic bacteria such as *Bacteroides fragilis* and other GI-tract microbes (Bhattacharjee and Lukiw, 2013; Foster et al., 2016; Zhao et al., 2017b,c; Cox and Weiner, 2018; Zhan et al., 2018; Zhao and Lukiw, 2018). The co-localization and eventual envelopment of sporadic AD-affected neuronal nuclei with a “clathrin-like” cage of GI-tract microbiome-derived LPS suggests that there exists a novel pathogenic contribution of LPS to neuron-specific gene expression and transcriptional output from AD-affected neurons (Zhao et al., 2017b). In agreement with previous reports a remarkably high proportion of the LPS signal in AD neocortex and hippocampus and in LPS-treated HNG cells are associated with neuronal nuclei (Figures 1–5 and Supplementary File 1; Zhao et al., 2017a,b,c; Zhao and Lukiw, 2018). Recent data further indicates that patients with moderate-to-advanced sporadic AD appear to have a significantly higher population of LPS-enveloped nuclei, and a correspondingly lower amount of NF-L associated with that region of the AD brain (Zhao et al., 2017b; Zhao and Lukiw, 2018) (Figures 5, 6).

In conclusion, the current work provides five novel observations: (i) that in AD neocortex LPS has a remarkable biophysical affinity for neuronal nuclei; (ii) that this action appears to selectively impair the transcriptional abundance of neuron-specific elements such as NF-L, known to be normally required for the maintenance of neuronal cytoarchitecture, synaptic connections, and the homeostatic signaling operations of neurons; (iii) that GI tract microbiome-derived neurotoxins may contribute to AD-type pathological and neuronal architectural change; (iv) that LPS-treated HNG brain cells in primary culture can recapitulate this phenomenon both at the biophysical and transcriptional level; and (v) perhaps most significantly, that LPS-treated HNG cells in primary culture can provide a highly useful experimental platform for further study on LPS effects on AD-like processes and their pathogenic consequences. These results also support the hypothesis that GI-tract derived, microbial neurotoxins such as LPS affect the efficient readout of AD-relevant neuronal-specific genetic information, such as that from the NF-L gene, and progressively contribute to cytoarchitectural aberrations, neuronal atrophy, and synaptic disorganization all of which are characteristic features of the sporadic AD brain (Figure 6).

## Ethics Statement

Procedures involving short post-mortem interval (PMI) human tissues and human brain primary cell cultures (HNG) were followed and handled in strict accordance with the ethics review board policies at donor institutions, and the Institutional Biosafety Committee/Institutional Review Board (IBC/IRB) ethical guidelines at the LSU Health Sciences Center, LA 70112, United States (IBC No. 12323; IRB No. 6774).

## Author Contributions

LC, VJ, WL, and YZ preformed the experiments and analyzed the data. WL wrote the paper.

## Conflict of Interest Statement

The authors declare that the research was conducted in the absence of any commercial or financial relationships that could be construed as a potential conflict of interest.

## Footnotes

1 https://www.mikroskop.com.pl/pdf/LSM700_1.pdf

2 https://www.sigmaaldrich.com

3 http://www.abcam.com/e-coli-lps-antibody-ab211144.html

4 https://imagej.net/Fiji/Downloads
