# Supplementary material for: Microbiome-Derived Lipopolysaccharide (LPS) Selectively Inhibits Neurofilament Light Chain (NF-L) Gene Expression in Human Neuronal-Glial (HNG) Cells in Primary Culture
Source: Front Neurosci. 2018 Dec 5;12:896. doi: 10.3389/fnins.2018.00896 (PMC6289986; doi:10.3389/fnins.2018.00896)
Supplement: Supplementary file 1 [file Data_Sheet_1.PDF]

Supplementary file 1

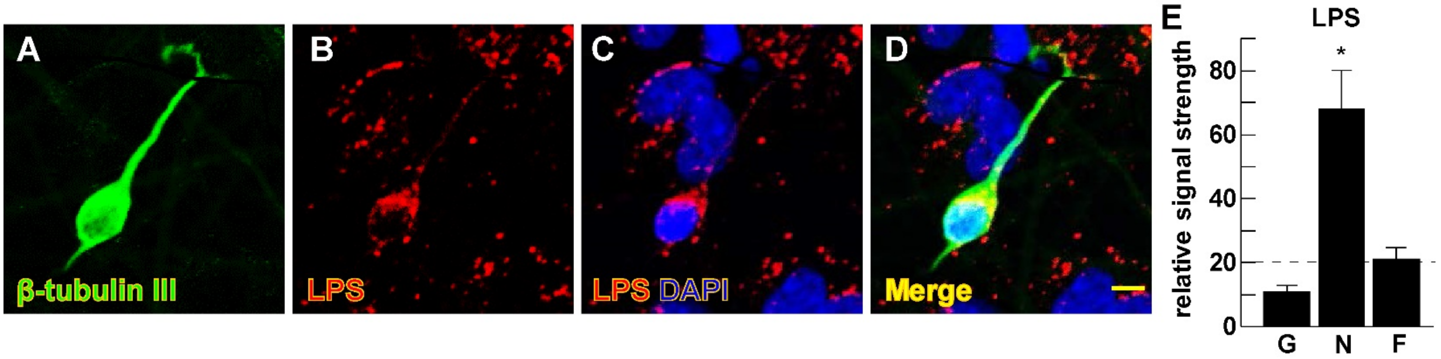

Supplementary file 1 - **Details of accumulation of LPS in HNG cells and their nuclei in primary culture** - (**A**) β-tubulin III (βTUBIII; neuron-specific) staining of HNG cells (green stain; λ_{max} = 520 nm); **(B)** LPS staining of HNG cells (red stain; λ_{max} = 690 nm); **(C)** LPS-DAPI staining of HNG cells and nuclei (blue nuclear stain; λ_{max} = 470 nm); and **(D)** merge of βTUBIII, LPS and DAPI signals; only two types of cells – neurons and astroglia – predominate in HNG cell cultures (**Fig 4A**; Lukiw et al., 2005); in these HNG cells, total LPS signal minus neuronal-nuclei associated LPS = glial-nuclei associated LPS or free LPS; **(E)** bar graph of quantitation of association of LPS with glial nuclei (**G**), neuronal nuclei (**N**) or free LPS (**F;** non-nuclei associated) using ImageJ software (NIH) and as described in the '***'Materials and Methods Section - Antibodies - Specificity and Validation***' section of this paper; in HNG cells added LPS was associated with neuronal (**N**) nuclei almost 7-fold that of LPS-association with glial (**G**) nuclei; there was more free LPS (**F**; not associated with any nuclear type) than associated with **G**; this suggests the preferential association of LPS with neuronal nuclei in HNG cells under the conditions specified; confocal images of HNG cells differentiated from human neural progenitor cells were cultured for 2 weeks followed by treatment with 20 ng/ml of LPS for 48 hr; LPS co-staining with neuronal marker β-TUBIII shows accumulation of LPS inside neurons and surrounding nuclei; the morphology of LPS inside cells resembles those seen in AD brain tissues; the association of LPS with neuronal cells or nuclei is highly significant; scale bar = 5 μm; horizontal dashed bar at 20 included for ease of comparison; N= 3 to 5; ***p**<0.01 (ANOVA).
